# Supplementary material for: Walk Locomotion Kinematic Changes in a Model of Penetrating Hippocampal Injury in Male/Female Mice and Rats
Source: Brain Sci. 2023 Nov 2;13(11):1545. doi: 10.3390/brainsci13111545 (PMC10669690; doi:10.3390/brainsci13111545)
Supplement: Supplementary file 1 [file brainsci-13-01545-s001.zip › brainsci-2664501-supplementary.pdf]

Supplementary Figure S1

We compared the displacement curves and calculated the dissimilarity factor between them using the Euclidean distance between each of the points of the normalized curve on the horizontal (X) and vertical (Y) axes (**A** and **B**). X_a (*i*) − X_b (*i*) is the difference (*d*) between the coordinates in X, and “Y_1 (*i*) − Y_2 (*i*)” in y of every point in the graph when comparing two steps (a and b); and “*i*" is the percent in the step cycle.

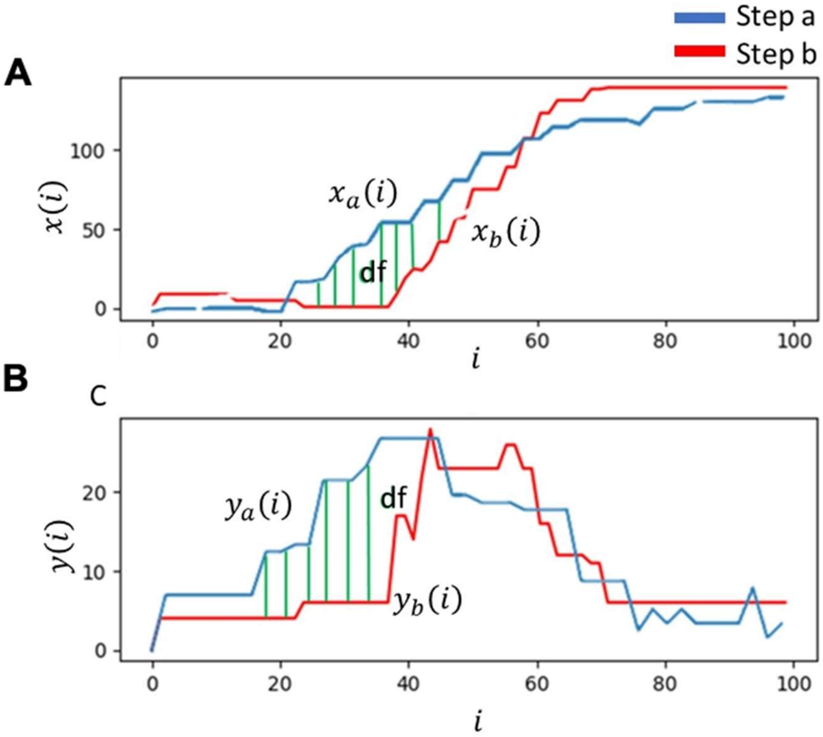

Supplementary Figure S2

It shows a table with the total points statistically significant in the step cycle in VD analysis of all rats/mice, male/female groups per side.

			Side	
		**C**ontrol vs **L**esioned	L	R
Metatarsus	**R**at	CMR vs LMR	35	9
		CFR vs LFR	0	8
	**M**ice	CMM vs LMM	8	6
		CFM vs LFM	14	0
Ankle	**R**at	CMR vs LMR	63	62
		CFR vs LFR	10	22
	**M**ice	CMM vs LMM	18	6
		CFM vs LFM	16	2
Knee	**R**at	CMR vs LMR	9	4
		CFR vs LFR	8	42
	**M**ice	CMM vs LMM	0	4
		CFM vs LFM	22	6

**M**ale
**F**emale
